# In Vitro–In Vivo Correlation of Posaconazole–Amphotericin B Combination against *Candida albicans*: In Vitro Interacting Concentrations Are Associated with In Vivo Free Drug Levels

**DOI:** 10.3390/jof9040434

**Published:** 2023-04-01

**Authors:** Joseph Meletiadis, Maria-Ioanna Beredaki, Antigoni Elefanti, Spyros Pournaras, Anouk Muller

**Affiliations:** 1 Clinical Microbiology Laboratory, Attikon University Hospital, Medical School, National and Kapodistrian University of Athens, 12462 Athens, Greece; 2 Department of Medical Microbiology and Infectious Diseases, Erasmus MC, 3015 CN Rotterdam, The Netherlands; 3 Department of Medical Microbiology, Haaglanden Medisch Centrum, 2512 VA The Hague, The Netherlands

**Keywords:** amphotericin B, posaconazole, combination therapy, *Candida albicans*, in vitro–in vivo correlation

## Abstract

The in vitro/in vivo correlation of antifungal combination testing is necessary in order to assess the efficacy of combination regimens. We, therefore, attempted to correlate in vitro chequerboard testing of posaconazole (POS) and amphotericin B (AMB) with the in vivo outcome of combination therapy against experimental candidiasis in a neutropenic murine model. The AMB + POS combination was tested against a *Candida albicans* isolate. In vitro, a broth microdilution 8 × 12 chequerboard method with serial two-fold drug dilutions was used. In vivo, CD1 female neutropenic mice with experimental disseminated candidiasis were treated with i.p. AMB and p.o. POS alone and in combination at three effective doses (ED20, ED50 and ED80 corresponding to 20%, 50% and 80% of maximal effect, respectively). CFU/kidneys after 2 days were determined. The pharmacodynamic interactions were assessed based on Bliss independence interaction analysis. In vitro, a Bliss antagonism of −23% (−23% to −22%) was observed at 0.03–0.125 mg/L of AMB with 0.004–0.015 mg/L of POS, while a Bliss synergy of 27% (14%–58%) was observed at 0.008–0.03 mg/L of AMB with 0.000015–0.001 mg/L of POS. In vivo, Bliss synergy (13 ± 4%) was found when an AMB ED20 of 1 mg/kg was combined with all POS ED 0.2–0.9 mg/kg, while Bliss antagonism (35–83%) was found for the combinations of AMB ED50 2 mg/kg and ED80 3.2 mg/kg with POS ED80 of 0.9 mg/kg. Free drug serum levels of POS and AMB in in vivo synergistic and antagonistic combinations were correlated with the in vitro synergistic and antagonistic concentrations, respectively. Both synergistic and antagonistic interactions were found for the AMB + POS combination. POS compromised the efficacy of high effective AMB doses and enhanced low ineffective AMB doses. In vitro concentration-dependent interactions were correlated with in vivo dose-dependent interactions of the AMB + POS combination. In vivo interactions occurred at free drug serum levels close to in vitro interacting concentrations.

## 1. Introduction

Combination therapy is often employed in order to manage infections by resistant isolates, sterilize difficult-to-treat sites, overcome subtherapeutic drug levels and broaden the antifungal spectrum [1]. Although combination therapy can increase fungicidal efficacy, reduce toxicity and prevent the emergence of fungal resistance, detrimental effects may occur in the case of antagonistic interactions, where the effect of combined drugs is smaller than the effect of the drugs alone. There are several methods for in vitro combination testing, with the broth microdilution chequerboard method being the most commonly used [2,3]. Although it provides information about interactions at several concentrations, it is unknown how these interactions are correlated with in vivo outcomes and whether in vitro concentrations are associated with in vivo drug levels. An in vitro test that could predict in vivo outcomes would be of major importance since it could be used to guide antifungal combination therapy, determine patients that will benefit from combination therapy and optimize doses in order to enhance synergistic interactions and avoid antagonistic combinations. Combination therapy of azoles with amphotericin B has been extensively studied in vitro and in animals, with conflicting results ranging from synergistic to antagonistic effects, whereas no effect has been found in clinical studies [2]. As both synergistic and antagonistic interactions have been previously described for the combination of triazoles with amphotericin B, such combinations pose a real challenge in in vitro–in vivo correlation studies.

We, therefore, investigated the in vitro and in vivo interaction between amphotericin B and posaconazole against *Candida albicans* and attempt to link in vitro interacting concentrations with in vivo drug levels. 

## 2. Materials and Methods

***Candida* isolate.** One wild-type (WT) clinical *C. albicans* strain isolated from a patient with disseminated candidiasis and identified with MALDI-TOF and VITEK system was used for the in vitro and the in vivo experiments. The amphotericin B and posaconazole CLSI [4] minimal inhibitory concentrations (MICs) were 0.125 mg/L and 0.004 mg/L, respectively. The isolate was stored in normal sterile saline with 10% glycerol at −70 °C; 24 h prior to study, the organism was revived by subculturing on Sabouraud dextrose agar (SDA) plates supplemented with gentamicin and chloramphenicol (SGC2, Biomerieux) to ensure purity and viability. Inocula suspensions of the subcultured yeast were prepared in sterile normal saline and adjusted after counting in a Neubauer hemacytometer to a final inoculum of 2.5 × 10^3^ CFU/mL, for the in vitro experiments, and 10^6^ CFU/mL, for the in vivo experiments. The CFU number was confirmed by quantitative cultures on SDA plates.

**Antifungal drugs and medium.** Posaconazole (POS; Merck Greece, Athens, Greece) and amphotericin B (AMB; Sigma Aldrich, Athens, Greece) were supplied as pure powders for the in vitro experiments. Stock solutions of 10 mg/mL for posaconazole and 5 mg/mL for amphotericin B were prepared in sterile dimethyl sulfoxide (DMSO; CarloErbaReactifs-SDS, Val de Reuil, France) and stored at −70 °C until use. For the in vivo experiments, posaconazole was obtained as a 40 mg/mL clinical suspension (Noxafil, Merck Greece, Athens, Greece), and for amphotericin B, a clinical formulation was used (Fungizone, Bristol-Myers Squibb, Princeton, NJ, USA), reconstituted according to manufacturer’s instructions. RPMI 1640 medium with L-glutamine and without bicarbonate buffered to pH 7.0 with 0.165M MOPS was used as the growth medium for the in vitro experiments.

**In vitro combination testing.** The in vitro interactions between POS and AMB were studied using a two-dimensional (8 × 12) checkerboard microdilution method in sterile 96-well microtitration plates, in accordance with CLSΙ M27-A3 standard methodology [4]. The antifungal agents were prepared in serial two-fold dilutions and ranged from 1.5 × 10^−5^ to 0.016 mg/L for posaconazole and 0.008 to 0.5 mg/L for amphotericin B. Aliquots of 50 μL of each drug, at concentrations four times the final concentration, were added in the wells of the 96-well plates. Plates were stored at −70 °C until the day of the experiment. On the day of the experiment, plates were thawed, inoculated with 100 μL of the *C. albicans* suspension and incubated for 24 h at 37 °C. Growth in each well was quantified spectrophotometrically, in which the optical density (OD) at 630 nm of each well was measured. The percentage of growth in each well was calculated as the OD of each well/OD of the drug-free well after subtracting the background OD obtained from broth-inoculated microtiter plates processed in the same manner as the conidia-inoculated plates. The MIC endpoints corresponded to either complete (>90% growth inhibition for amphotericin B) or prominent (>50% growth inhibition for posaconazole) decrease in turbidity compared to turbidity in growth control well. All experiments were performed in triplicate. 

**Animals.** Four- to six-week-old CD1 female mice weighing 23–27 g were used. The animals were allowed to acclimatize for at least 5 days upon arrival and were housed under standard conditions with drink and feed supplied ad libitum. All animal procedures were carried out in the Animal facility for Medical and Scientific Purposes at the University Hospital Attikon (EL 25 BIO 014), Athens, Greece. All animal studies were conducted in accordance with the recommendations of the European Community (Directive 86/609/EEC, 24 November 1986, and 2010/63/EE, 2010 (276/33/20/10.2010)). The studies were approved by its Animal Welfare Committee no K/7391/2010.

**Infection model.** Mice were rendered neutropenic by injecting cyclophosphamide (Mead Johnson Pharmaceuticals, Evansville, IN, USA.) subcutaneously for 4 days (150 mg/kg of body weight) and at 1 day (100 mg/kg) before infection so that neutrophil counts remained below 100/mm^3^ throughout the study. Disseminated infection with the *Candida* organism was achieved by injection of 0.1 mL of inoculum (10^6^ CFU/mL), 2 h prior to start of drug therapy. At the end of the study period, animals were sacrificed, and their kidneys were immediately removed and placed in sterile saline at 4 °C. The homogenates were serially diluted at 1:10 and 1:100, and aliquots were placed on SDA plates for viable fungal colony counts after incubation for 24 h at 30 °C. The lower limit of detection was 40 CFU/kidney. Results were expressed as the mean CFU/kidney for two mice (four kidneys). 

**Treatment**. Posaconazole and amphotericin B doses were administered every 24 h for the 48 h study period. Groups of two mice were used for each dosing regimen. At the end of study, mice were euthanized, and their kidneys were immediately processed for CFU determinations. In pilot experiments, posaconazole was orally administered at doses ranging from 0.03 to 32 mg/kg, while amphotericin B alone was administered intraperitoneally at doses ranging from 0.31 to 5 mg/kg in 0.1 mL volumes. A sigmoid dose-effect model was used to measure the in vivo potency of posaconazole and amphotericin B by nonlinear regression analysis (E_max_ model), described by the equation E = E_max_ × (D/ED_50_)^m^/[1 + (D/ED_50_)^m^], where E is the CFU/kidney observed at a certain dose, D, of antifungal agent, Ε_max_ is the CFU/kidney obtained from the control group, ED_50_ is the dose corresponding to 50% of Ε_max_ and m is the slope of the dose/response curve (Hill slope). Doses required to produce 20% (ED20), 50% (ED50) and 80% (ED80) of the maximal effect were also calculated. Consequently, the therapeutic effect of all possible posaconazole and amphotericin B combinations (3 × 3) plus the monotherapy regimens were studied. All data were analyzed using the statistics software package GraphPad Prism, version 5.0, for Windows (GraphPad Software, San Diego, CA, USA). 

**In vitro–in vivo correlation.** Amphotericin B and posaconazole mouse serum concentrations were predicted based on previously published pharmacokinetic studies using the same mouse strain [5,6]. Amphotericin B and posaconazole doses were correlated with Cmax and Cmin concentrations by non-linear regression analysis using one-phase association equation C = Plateau ∗ (1 − e^−k*Dose^), where C is the peak concentration (Cmax) or trough concentration (Cmin) of amphotericin B or posaconazole in mouse serum in mg/L, Plateau is the C at infinite Dose, K is a constant and Dose is the dose of amphotericin B or posaconazole in mg/kg. Cmax and Cmin concentrations of ED20, ED50 and ED80 of the present study were then estimated via interpolation. Free drug levels were determined based on 95% and 99% protein binding for amphotericin B and posaconazole, respectively, in mouse serum [5,6]. 

**Pharmacodynamic drug interaction analysis.** The in vitro and in vivo drug interactions were analyzed using Bliss independence theory, described by the equation I_IND_ = I_AMB_ + I_POS_ − I_AMB_ × I_POS_ (1), for every posaconazole and amphotericin B combination, where I_AMB_ and Ι_POS_ correspond to the percentage of growth inhibition caused by amphotericin B and posaconazole acting alone, and I_IND_ is the theoretical percentage of growth inhibition caused by a certain noninteractive (independent) combination. This equation is equal to E_IND_ = *E*_AMB_ × E_POS_ (given that E = 100% − I), where *E*_AMB_ and E_POS_ are the experimentally determined percentages of fungal growth (as calculated from the absorbance values for the in vitro experiments or from the CFU/kidney values for the in vivo experiments) for amphotericin B and posaconazole, respectively, in monotherapies, and *E*_IND_ is the theoretical percentage of fungal growth if amphotericin B and posaconazole were acting independently. The difference (ΔΕ) between the theoretical (E_IND_) and the experimentally determined (*E*_EXP_) percentage of fungal growth was calculated, and its statistical significance was assessed by Student’s *t*-test. When *E*_EXP_ was statistically significantly higher or lower than *E*_IND_ (positive or negative ΔE, respectively), statistically significant synergy or antagonism was concluded. In any other case, Bliss independence was assumed.

For the in vitro experiments, the ΔΕ was calculated for all combinations of the checkerboard at different concentrations of the 2 drugs. A three-dimensional interaction surface (ΔΕ on Z-axis at each concentration of the two drugs on X- and Y-axis) was constructed with synergistic and antagonistic interactions above and below the 0-plane, respectively.

## 3. Results

**Figure 1:**
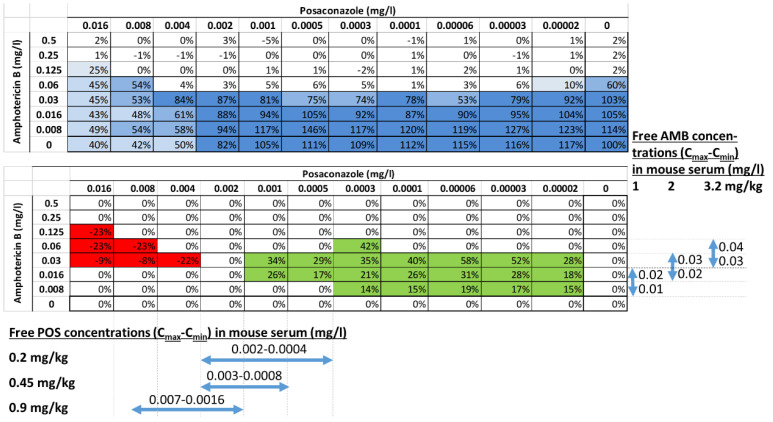
Posaconazole–amphotericin B in vitro interaction based on Bliss independence theory and posaconazole–amphotericin B free concentrations in mouse serum. The top panel depicts the percentage of fungal growth, while the bottom panel depicts statistically significant synergy (green color) or antagonism (red color) at each concentration of posaconazole and amphotericin B alone and in combination. The predicted free posaconazole and amphotericin B Cmax and Cmin concentrations in mouse serum of 3 × 3 dosing regimens used in combination therapy are also shown.

**Figure 2:**
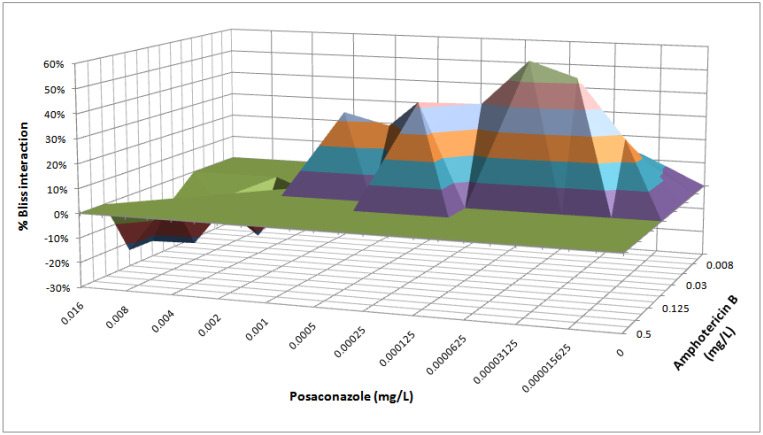
Interaction surface obtained from response surface analysis of Bliss-independence-based drug interaction model for the in vitro combination of amphotericin B and posaconazole against *Candida albicans.* The zero plane indicates Bliss-independent interactions, whereas values below the zero plane indicate statistically significant antagonistic interactions (negative ΔΕ ). The different tones in the 3-dimensional plot represent different percentile bands of antagonism.

**In vitro combination experiments.** The results of Bliss independence drug interaction analysis for the in vitro pharmacodynamic interactions of posaconazole and amphotericin B are summarized in Figure 1. Bliss antagonism was found (ΔΕ −23% to −8%) for the combination of amphotericin B 0.03–0.125 mg/L with posaconazole 0.004–0.016 mg/L, whereas Bliss synergy (ΔΕ 14% to 58%) was found for amphotericin B of 0.008 to 0.06 mg/L with posaconazole 0.00002–0.001 mg/L. The 3D interaction surface (Figure 2) revealed synergy at low posaconazole concentrations, while antagonism occurred at higher posaconazole concentrations, with amphotericin B concentrations of synergistic interaction being lower than amphotericin B concentrations of antagonistic interactions (median 0.016–0.03 vs. 0.03–0.125 mg/L, respectively).

**Figure 3:**
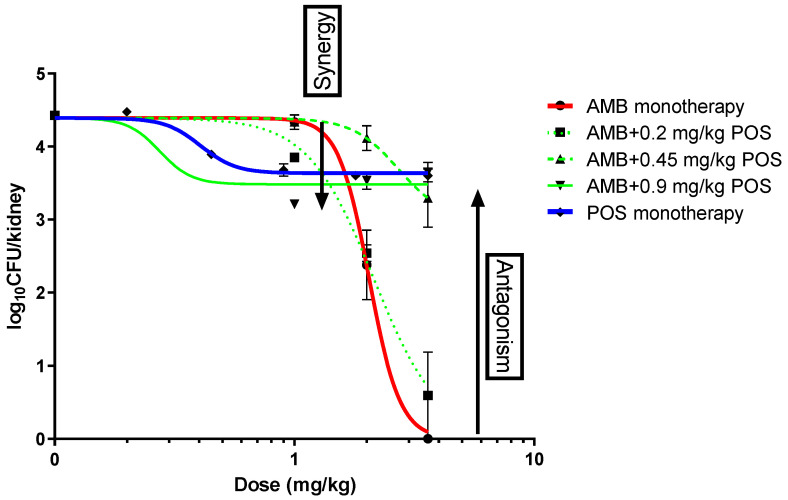
In vivo dose–response curves for amphotericin B (AMB), posaconazole (POS) alone and in combination. Synergy was determined at lower amphotericin B doses combined with posaconazole, while antagonism occurred at higher amphotericin B doses in combination with posaconazole.

**Figure 4:**
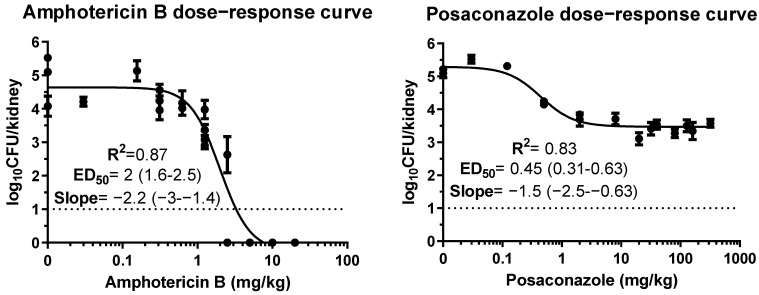
In vivo dose–response curves of amphotericin B and posaconazole monotherapy. Error bars correspond to SD.

**In vivo combination experiments.** Dose–response curves of amphotericin B and posaconazole monotherapy were described well by the Emax model, with fungal kidney burden ranging from 4.5 to 5.5 log_10_CFU/kidney in placebo to 0 log_10_CFU/kidney at high AMB doses to 3.5 log_10_CFU/kidney at high posaconazole doses (Figure 3). The ED_20_, ED_50_ and ED_80_ were 1, 2 and 3.6 mg/kg for amphotericin B and 0.2, 0.45 and 0.9 mg/kg for posaconazole. Amphotericin B led to complete clearance of the infection in the kidneys when administered in higher doses. Figure 4 shows the sigmoid dose–response curves for monotherapies as well as combination therapy.

As far as the combination therapy is concerned, two separate phenomena took place. First, when lower amphotericin B doses were combined with effective doses of posaconazole, fungal burden was lower compared to amphotericin B monotherapy, indicating synergy. On the other hand, when higher amphotericin B doses were combined with posaconazole, the fungal burden was higher compared to amphotericin B monotherapy, indicating antagonism.

The results of the Bliss independence drug interaction analysis for the in vivo pharmacodynamic interaction are summarized in Table 1. The percentage of fungal burden as determined for each dose of posaconazole and amphotericin B alone and in combination is reported. Regarding the monotherapies, the analysis showed that fungal burden was reduced compared to controls by 16 ± 3% and 100% at the highest doses of posaconazole (0.9 mg/kg) and amphotericin B (3.6 mg/kg), respectively. Statistically significant Bliss synergy was observed for combinations of the lowest amphotericin B dose of 1 mg/kg with the lowest posaconazole dose of 0.2 mg/kg (13 ± 4%) and the highest posaconazole dose of 0.9 mg/kg (10 ± 4%), while Bliss antagonism was found for a combination of the highest and intermediate amphotericin B doses of 3.2 and 2 mg/kg with the highest and intermediate posaconazole doses of 0.9 and 0.45 mg/kg (35–83%).

**Figure 5:**
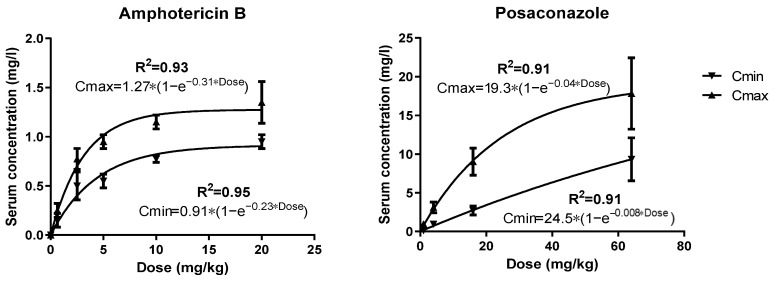
Dose–serum concentrations relationship for amphotericin B and posaconazole in mice.

**Correlation of in vitro–in vivo concentrations**. One-phase association model described well (R^2^ > 0.90) the dose–serum concentration relationships of amphotericin B and posaconazole (Figure 5). Based on these relationships, free amphotericin B and posaconazole Cmax and Cmin concentrations of 1, 2 and 3.2 mg/kg of amphotericin B and 0.2, 0.45 and 0.9 mg/kg of posaconazole doses were calculated and are shown in Table 1. Free drug serum concentrations of the highest doses of posaconazole 0.9 mg/kg and amphotericin B 3.2 mg/kg correlated with in vitro concentrations where most antagonistic interactions occurred (0.004–0.008 mg/L of posaconazole and 0.03–0.06 mg/L of AMB), whereas free drug serum concentrations of the lowest doses of posaconazole 0.2 mg/kg and amphotericin B 1 mg/kg correlated with in vitro concentrations where mostly synergistic interactions occurred (0.0005–0.001 mg/L of posaconazole and 0.008–0.0016 mg/L of amphotericin B).

## 4. Conclusions

In vitro pharmacodynamic studies of drug combinations are a valuable tool for exploring interactions at different concentrations and detecting synergistic and antagonistic effects. However, the correlation of in vitro experiments with in vivo results is difficult because in vitro interactions are usually summarized with a unique index (e.g., the FIC index) or assessed at specific concentrations (e.g., in time–kill assays), which does not describe the interactions at different concentrations. More importantly, it is unknown how to correlate in vitro concentrations with in vivo drug levels. In the present study, we correlated the in vitro combination results of posaconazole and amphotericin B with the in vivo effects observed during combination therapy in a murine model of disseminated candidiasis and linked in vitro interacting concentrations with in vivo free drug serum levels. Bliss response surface analysis revealed in vitro and in vivo dose/concentration-dependent interactions of posaconazole–amphotericin B combinations, with synergy found at lower ineffective doses/concentrations of amphotericin B and antagonism at higher effective doses/concentrations of amphotericin B. The in vivo free drug serum levels of synergistic and antagonistic combinations in the animal model correlated with the in vitro concentrations of synergistic and antagonistic combinations in the checkerboard method, respectively.

Most in vitro combination studies of azoles with amphotericin B resulted in additive/indifferent interactions and sometimes exhibited antagonism when assessed with the FIC index [7]. In order to capture the one-two-fold dilution error of MICs, a wide range of FICi from 0.5 to 4 was proposed for defining additivity/indifference [8], thus detecting only very strong synergistic and antagonistic interactions with FICi ≤0.5 and ≥4, i.e., at least two two-fold dilutions of MIC decrease and increase, respectively. We have previously shown that variability in the checkerboard method may be low since MICs of drugs alone and in combination are determined at the same time, and therefore, a narrower FICi range of additivity/indifference can be used in order to capture significant interactions [9,10]. Another important drawback of FICi is that interactions are usually assessed at the MIC level, ignoring interactions at lower sub-MIC concentrations. The posaconazole–amphotericin B combination will be deemed additive based on the complete growth inhibition endpoint of amphotericin B and the antagonistic based partial growth inhibition endpoints of posaconazole, with FICis of 0.5007 and 4, respectively. However, Bliss analysis identified both synergistic and antagonistic interactions at different concentrations. The same conclusion could be drawn using a narrower additivity/indifference FICi range of 1–1.25, as previously suggested [9,10]. The detection of pharmacodynamic interactions with Bliss analysis and not with the FIC index analysis has also been reported in previous studies for invasive aspergillosis and candidiasis [11,12]. More importantly, the concentrations corresponding to both FICis of complete and partial growth inhibition endpoints were different than the serum drug levels of the in vivo combination regimens used in the present study and, thus, could not be used to predict in vivo outcomes. Response surface models based on either Loewe additivity or Bliss independence theory can be used to describe the interactions at different drug concentrations and facilitate in vitro–in vivo correlations and extrapolations to humans [13,14].

In vivo interactions between azoles and amphotericin B against *Candida* species have been studied by several groups. In models of systemic candidiasis with *Candida albicans*, Louie et al. observed antagonism between the triazole fluconazole and amphotericin B [15], while Sugar and Liu also reported antagonism between itraconazole and amphotericin B [16]. Moreover, Louie et al. reported that fluconazole also exhibited antagonism in experimental rabbit models for endocarditis and pyelonephritis against *Candida albicans* [17]. However, Sanati et al. found no antagonism between fluconazole and amphotericin B against *Candida albicans* in experimental models of candidiasis in neutropenic mice and endocarditis in rabbits [18]. This may be due to the different doses studied in each case. Amphotericin B is usually administered at high effective doses, which are associated with antagonism, as was found in the present study. Synergy between posaconazole and amphotericin B was found in an experimental model of invasive candidiasis by *C. albicans*, where low AMB doses (0.5–1 mg/kg) associated with 50% survival were used [19], thus, supporting the results of the present study, where synergy was found at similar amphotericin B doses that do not elicit a maximal effect. Both synergistic and antagonistic interactions have been previously described for the combination of triazoles with amphotericin B [13,20].

Although the molecular mechanism that describes the concentration-dependent interaction between amphotericin B and posaconazole is unknown, this effect could be related to the model of action of amphotericin B, as originally proposed by Cohen et al. [21]. According to this model, amphotericin B at low concentrations (0.2–0.8 μM) forms precursor non-hydrophilic pore-like structures on the surface of fungal membranes resembling ion channels, without the direct involvement of ergosterol molecules. These structures increase the permeability of membranes to urea and glucose molecules. At higher concentrations (>1.2 μM), the initially formed structures interact further with the molecules of ergosterol, creating hydrophilic pores characterized by a large diameter. The synergistic interaction between amphotericin B and posaconazole at the area of lower concentrations of amphotericin B could be explained by changes in the permeability of the fungal membrane induced by amphotericin B, followed by the increased inflow or insufficient outflow of posaconazole molecules to or from the cell, respectively. Given the fact that ergosterol is not involved in the formation of these precursor structures of amphotericin B, the inhibition of the biosynthetic pathway of ergosterol by posaconazole will not antagonize the effect of amphotericin B [20]. On the other hand, higher concentrations of amphotericin B form hydrophilic pores, a process in which the involvement of ergosterol molecules is necessary, and for this reason, the inhibition of the biosynthesis of ergosterol caused by posaconazole will antagonize the activity of amphotericin B.

The critical question is which of all these interactions are clinically relevant. One point that should be emphasized is that a synergistic combination may not necessarily be the most effective regimen, as the effect elicited by a higher dose of one drug could be higher than the effect of lower doses in a synergistic combination of two drugs. However, higher doses are usually associated with toxicity, particularly for amphotericin B, and therefore, a lower amphotericin B dose in combination with a second drug could produce the same effect as a high dose of amphotericin B while minimizing toxicity. In our model, the most effective regimen was amphotericin B alone at the highest dose, as this was the only regimen that cleared fungi in the kidney. However, the clearance of fungi may not be the clinically relevant pharmacodynamic target. A 50% of maximal effect and stasis were proposed for triazoles and echinocandins, respectively [22]. For amphotericin B, the clinically relevant pharmacodynamic target is not clear, but we could assume that it is stasis as echinocandins since both drug classes are fungicidal against *Candida* spp. Previous PK/PD studies showed that stasis is achieved at ~Cmax/MIC 4 (fCmax/MIC 0.2) [5], which is close to the predicted PK/PD index of the present study since stasis was found at the dose of 2 mg/kg (Cmax 0.6 mg/L) for a *C. albicans* isolate with MIC 0.125 mg/L (i.e., 0.6/0.125 = 4.8 Cmax/MIC). Similarly, for posaconazole, a 50% of maximal effect was previously found to correspond to 17 (6.12–26.7) fAUC/MIC [23], which is close to the corresponding predicted fAUC/MIC of 6.5 in the present study (fAUC_0–24_ of ED_50_ 0.45 mg/kg, is ~0.026 mg.h/L and the MIC of the *C. albicans* isolate is 0.004 mg/L, i.e., 0.026/0.004 = 6.5). Finally, in order to answer the previous question on the clinical relevance of the pharmacodynamic interactions, one should consider the drug levels where interactions occurred in relation to the pathogen’s MIC and whether they are achievable with human doses. The standard dose of amphotericin B of 1 mg/kg results in mean ± SD Cmax 2.83 ± 1.17 mg/L (fCmax 0.14 ± 0.06 mg/L) [24], indicating that, in most patients, amphotericin B will not attain the PK/PD target of fCmax/MIC 0.2 for WT *C. albicans* isolates with MICs up to the epidemiological cutoff value of 1 mg/L for amphotericin B and may benefit from combination therapy. For the few patients that will attain the PK/PD target of 0.2 fCmax/MIC, thus, amphotericin B will be effective alone, combination therapy with posaconazole will compromise this efficacy. Similarly, a posaconazole standard i.v. dose of 300 mg/d results in mean ± SD AUC_24_ 34.3 ± 13.6 mg.h/L (fAUC_24_ 0.34 ± 0.14 mg.h/L) [25], indicating that most patients will not attain the PK/PD target of 17 fAUC/MIC for WT *C. albicans* isolates with MIC up to the epidemiological cutoff value of 0.06 mg/L for posaconazole and may also benefit from combination therapy. This is in agreement with clinical cases, where a favorable outcome is most commonly observed when amphotericin B was combined with a triazole, usually fluconazole [7]. Guidelines indicate that combination can be considered as an option particularly in severe deep-seated infections [26].

In conclusion, the present study demonstrated in vitro and in vivo concentration/dose-dependent interactions of posaconazole–amphotericin B combination. Synergy was observed when posaconazole was combined with low amphotericin B exposures, which were ineffective as monotherapy, whereas antagonism was found when posaconazole was combined with higher amphotericin B exposures, which were effective as monotherapy. Furthermore, the free drug serum levels of posaconazole and amphotericin B in synergistic and antagonistic combinations were correlated with the in vitro synergistic and antagonistic concentrations, respectively. This paper provides a framework to link in vitro interactive concentrations with in vivo exposures and combinations, determine patients that will benefit from combination therapy and optimize doses in order to enhance synergistic interactions, minimizing toxicity without compromising efficacy.

## Figures and Tables

**Table 1:** Results of Bliss-independence-based response surface analysis.

Dose (mg/kg)	Amphotericin B (Monotherapy) (E_EXP_)	0.2 mg/kg Posaconazole			0.45 mg/kg Posaconazole			0.9 mg/kg Posaconazole		
		E_EXP_ (%)	Ε_IND_ (%)	% ΔΕ (Bliss Interaction) *	E_EXP_ (%)	Ε_IND_ (%)	% ΔΕ (Bliss Interaction) *	E_EXP_ (%)	Ε_IND_ (%)	% ΔΕ (Bliss Interaction) *
0	101 ± 0	102 ± 1			89 ± 0			84 ± 2		
1	99 ± 3	87 ± 1	101 ± 3	13 (S)	99 ± 0	87 ± 2	−11 (A)	73 ± 2	83 ± 4	10 (S)
2	54 ± 15	58 ± 5	55 ± 13	−2 (I)	94 ± 6	48 ± 11	−45 (A)	80 ± 5	45 ± 12	−35 (A)
3.6	0 ± 0	13 ± 27	0 ± 0	−13 (I)	75 ± 18	0 ± 0	−75 (A)	83 ± 4	0 ± 0	−83 (A)

* (S: Bliss synergy, I: Bliss independence, A: Bliss antagonism).

## Data Availability

The data presented in this study are available on request from the corresponding author.
